# Supplementary material for: Multifunctional SERS Chip for Biological Application Realized by Double Fano Resonance
Source: Nanomaterials (Basel). 2024 Dec 19;14(24):2036. doi: 10.3390/nano14242036 (PMC11676296; doi:10.3390/nano14242036)
Supplement: Supplementary file 1 [file nanomaterials-14-02036-s001.zip › nanomaterials-3360092-supplementary.pdf]

Supplementary information

Multifunctional SERS Chip for Biological Application Realized by Double Fano Resonance

Weile Zhu [1], Huiyang Wang [2,3], Yuheng Wang [2,3], Shengde Liu [2,3,*], Jianglei Di [1] and Liyun Zhong [1]

1 Guangdong Provincial Key Laboratory of Photonics Information Technology, Guangdong University of Technology, Guangzhou 510006, China; 2112203214@mail2.gdut.edu.cn (W.Z.); jiangleidi@gdut.edu.cn (J.D.); zhongly@gdut.edu.cn (L.Z.)

2 School of Optoelectronic Science and Engineering, South China Normal University, Guangzhou 510006, China; 2021010155@m.scnu.edu.cn (H.W.); wangyh919@foxmail.com (Y.W.)

3 Guangdong Provincial Key Laboratory of Nanophotonic Functional Materials and Devices, South China Normal University, Guangzhou 510006, China

* Correspondence: liushengde@m.scnu.edu.cn

S1. The recurrence formula of reflectivity in the multi-layer film system

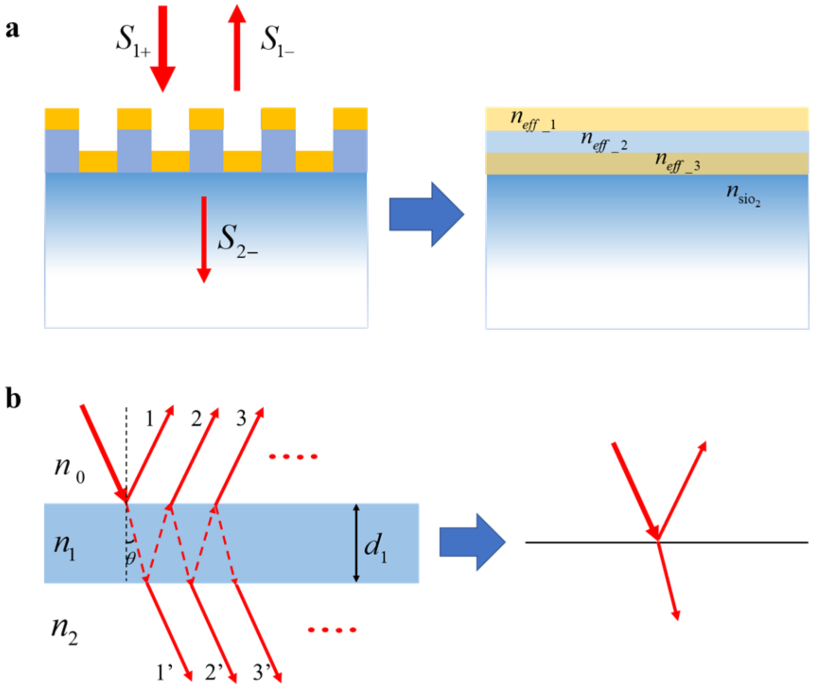

Figure S1. (a) Schematic diagram of the equivalent of the 3-layer nano-structured surface to 3 - layer thin film system; (b) schematic diagram of a film at different interfaces equivalent to an interface.

Considering the translation of SERS chip into 3-layer thin film system as shown in **Fig. S1a**, we first analyze the case of single-layer thin film, then generalize and apply the single-layer to the 3-layer film system, and get the analytical derivation of the reflectivity recursive formula (S3) in the main text.

As shown in **Figure S1b,** assumed that there is an isotropic thin film with index of n_1 and uniform geometric thickness of d_1 on the optical substrate with a refractive index of n_2, the refractive index of the incident medium is n_0, when the incident beam E_0 hits the upper surface of the film, the beam must be reflected on both surfaces of the film multiple times, resulting in a set of reflected beams $E_1, E_2, E_3 \cdots$, which can be express as:

$$\begin{cases} E_1 = r_{1+}E_0 \\ E_2 = t_{1+}r_{2+}t_{1-}e^{-j2\delta_1}E_0 \\ E_3 = t_{1+}r_{2+}r_{1-}r_{2+}t_{1-}e^{-j2\delta_1}E_0 \\ \cdots \end{cases} \tag{S1}$$

Here, $r_{1+}, r_{1-}, t_{1+}, t_{1-}, r_{2+}$ represent the reflection coefficient and transmission coefficient at the two interfaces respectively; subscript "+", "-" represent the energy direction of up or down, and "1" "2" represent the upper or lower side of thin films, $\delta_1 = (2\pi/\lambda)n_1 d_1 \cos\theta$ is the phase delay induced by the thin film, according to the Stokes' Law, we can get the relationship: $r_+ = -r_-$, $r_+^2 + t_+t_- = 1$, the total reflected amplitude can be expressed as:

$$E_R = \sum_{i=1}^{\infty} E_i = \left(r_{1+} + \frac{t_{1+}r_{2+}t_{1-}\mathbf{e}^{-j2\delta_1}}{1 - r_{2+}r_{1-}\mathbf{e}^{-j2\delta_1}} \right) E_0 = \frac{r_{1+} + r_{2+}\mathbf{e}^{-j2\delta_1}}{1 + r_{1+}r_{2+}\mathbf{e}^{-j2\delta_1}} E_0 \tag{S2}$$

The reflectivity of the single-layer film is:

$$r = \frac{E_R}{E_0} = \frac{r_1^+ + r_2^+ e^{-j2\delta_1}}{1 + r_1^+ r_2^+ e^{-j2\delta_1}} \tag{S3}$$

Formulations (S1)-(S3) show that two interfaces of the single-layer film can be replaced by an equivalent interface shown in **Fig. S1b**. Thus, the 3-layer film system can be replaced by one single interface gradually: first, starting from the bottom film adjacent to the substrate, the two interfaces of the bottom film can be equivalent to one interface, and then both the equivalent interface and the upper interface are equivalent to another new equivalent interface. By replacing all the films step by step, the recurrence formula of reflectivity in the multi-layer film system can be expressed:

$$r_{eff} = \frac{r_k + r_{k+1}\mathrm{e}^{-j2\delta_k}}{1 + r_k r_{k+1}\mathrm{e}^{-j2\delta_k}} \tag{S4}$$

Where r_k represents the equivalent reflectance coefficient of layer k, which obtained from the Fresnel formula, n_k represents the equivalent refractive index of the layer k, d_k represents the film thickness, and $\delta_k = (2\pi/\lambda)n_k d_k$ represents the phase delay introduced by the film.

S2. Analytical reflection model base on Couple Mode Theory

The derivation of the proposed analytical model described by formulation (S4) in main text is a multi-mode coupling based on Couple Mode Theory (CMT). To begin with, we defined a resonance system with an input port and an output port as shown in **Fig.S1a**. While an excitation light S_{1+} pumping the resonators in SERS chip, the resonator served as a linear system, is coupled to a partially reflection S_{1-} from port 1 and a partially transmission S_{2-} from port 2. Make sure that the complex amplitudes are normalized to the power per area: $S_{1+} = \frac{1}{2}\mathrm{Re}(E_{1+} \times H_{1+}^* \cdot \vec{z})$, then the system can be described by the scattering matrix if there is no loss and no other resonance inside the SERS chip:

$$\mathrm{s}_- = S\mathrm{s}_+ \tag{S5}$$

Here $s_- = \begin{pmatrix} s_{1-} \\ s_{2-} \end{pmatrix}$ represents the F-P mode, $s_+ = \begin{pmatrix} s_{1+} \\ s_{2+} \end{pmatrix}$ is incident beam and $S = \begin{pmatrix} S_{11} & S_{12} \\ S_{21} & S_{22} \end{pmatrix}$ is characterized by the Fresnel coefficients which are determined by effective medium and equivalent system mentioned above. Note that s_{2+} should be 0 as there is no light coming from bottom.

Suppose LSR and SPP-Bloch modes in the SERS chip are a unity whose complex amplitude is *u* for simplistic, according to the CMT, s_{1-} would be the result of coherent coupling of all the resonance modes including F-P resonance, LSR and SPP-Bloch once LSR and SPP are working. Thus (S5) can be rewrited as:

$$s_- = Ss_+ + Du \tag{S6}$$

where $D = \begin{pmatrix} d_{11} & d_{12} \\ d_{21} & d_{22} \end{pmatrix}$ is the coupling matrix, $u = \begin{pmatrix} u_1 \\ u_2 \end{pmatrix}$ are the complex amplitude of SLR mode and SPP-Bloch mode respectively.

To describe *u* in the resonance system, couple equation base on the perturbation theory is:

$$\frac{du_i}{dt} = \left(j\omega_i - \gamma_{i0} - \gamma_{i1} - \gamma_{i2} \right) u_i + \kappa_i s_{1+} \tag{S7}$$

Where γ_{i0} represents the loss induced by internal absorption of gold, γ_{i1} and γ_{i2} are the leaking rate of energy to upwards and downwards respectively and κ_i is the complex coupling coefficient from excitation light into the resonance modes in the SERS chip.

Consider the above discussion including (S6) and (S7), the reflectivity of the whole resonance system can be achieved:

$$\Gamma = \frac{s_{1-}}{s_{1+}} = S_{11} + \sum_{i=1}^{2} \frac{d_{1i}\kappa_i}{j\left(\omega - \omega_i\right) + \gamma_{i0} + \gamma_{i1} + \gamma_{i2}} \tag{S8}$$

Since the reflectivity in (S8) can not be larger than 1 in nature, that is $\Gamma \leq 1$. What's more, parameters in this formulation are constrained by some fundamental concepts, for example, the energy conservation in any physical system, time-reversal symmetry in Maxwell equation and reciprocity in scattering matrix:

$$\left|\kappa_i\right| = \sqrt{2(\gamma_{i1} + \gamma_{i2})} \Rightarrow \kappa_i = \sqrt{2(\gamma_{i1} + \gamma_{i2})} e^{j\theta_i} \tag{S9}$$

$$d_{1i} = \kappa_i^* \tag{S10}$$

By using these properties, it is possible to further refine the reflectivity in (S8):

$$\Gamma = \frac{s_{1-}}{s_{1+}} = S_{11} + \sum_{i=1}^{2} \frac{A_i e^{j\varphi_i}}{j\left(\omega - \omega_i\right) + \gamma_i} \tag{S11}$$

Where $\gamma = \gamma_{i0} + \gamma_{i1} + \gamma_{i2}$ is the total loss of the SERS chip, *A* is a parameter related to the leakage rate $\gamma_{i1} + \gamma_{i2}$, $\varphi_i = \phi_i + \theta_i$ is the total phase of resonance modes i and θ_i is the coupling phase of resonator and F-P resonance which is related to r_{eff} in (S3), ϕ_i is the phase of SPP-Bloch mode or SLR mode coupling to F-P mode.

By replacing the scattering matrix element S_{11} with r_{eff}, the reflectance of the SERS chip can be expressed:

$$R = \left| r_{\text{eff}} + \frac{A_1 e^{i\varphi_1}}{i\left(\omega - \omega_1\right) + \gamma_1} + \frac{A_2 e^{i\varphi_2}}{i\left(\omega - \omega_2\right) + \gamma_2} \right|^2 \tag{S12}$$
